# Effects of a liquefied petroleum gas stove and fuel intervention on head circumference and length at birth: A multi-country household air pollution intervention network (HAPIN) trial

**DOI:** 10.1016/j.envint.2024.109211

**Published:** 2025-01

**Authors:** Hina Raheel, Sheela Sinharoy, Anaité Diaz-Artiga, Sarada S. Garg, Ajay Pillarisetti, Kalpana Balakrishnan, Marilu Chiang, Amy Lovvorn, Miles Kirby, Usha Ramakrishnan, Shirin Jabbarzadeh, Alexie Mukeshimana, Michael Johnson, John P. McCracken, Luke P. Naeher, Ghislaine Rosa, Jiantong Wang, Joshua Rosenthal, William Checkley, Thomas F. Clasen, Jennifer L. Peel, Lisa M. Thompson, Household Air Pollution Intervention Network (HAPIN) investigators

**Affiliations:** a Nell Hodgson Woodruff School of Nursing, Emory University, Atlanta, GA, USA; b Hubert Department of Global Health, Rollins School of Public Health, Emory University, Atlanta, USA; c Center for Health Studies, Universidad del Valle de Guatemala, Guatemala City, Guatemala; d ICMR Center for Advanced Research on Air Quality, Climate and Health, Department of Environmental Health Engineering, Sri Ramachandra Institute for Higher Education and Research, Chennai, India; e Division of Environmental Health Sciences, University of California at Berkeley, Berkeley, CA, USA; f Biomedical Research Unit, Asociación Benéfica PRISMA, Lima, Peru; g Gangarosa Department of Environmental Health, Rollins School of Public Health, Emory University, Atlanta, CA, USA; h Department of Global Health and Population, Harvard T. H. Chan School of Public Health, Harvard University, Boston, MA, USA; i Department of Biostatistics and Bioinformatics, Rollins School of Public Health, Emory University, Atlanta, GA, USA; j Eagle Research Centre, Kigali, Rwanda; k Berkeley Air Monitoring Group, Berkeley, CA, USA; l Department of Environmental Health Science, College of Public Health, University of Georgia, Athens, GA, USA; m Faculty of Infectious and Tropical Diseases, London School of Hygiene and Tropical Medicine, London, UK; n Division of Epidemiology and Population Studies, Fogarty International Center, National Institutes of Health, Bethesda, MD, USA; o Division of Pulmonary and Critical Care, School of Medicine, Johns Hopkins University, Baltimore, MD, USA; p Center for Global Non-Communicable Disease Research and Training, School of Medicine, Johns Hopkins University, Baltimore, MD, USA; q Department of Environmental and Radiological Health Sciences, Colorado State University, Fort Collins, CO, USA

**Keywords:** Birth length, Head circumference, Household air pollution, LPG stove, Intervention

## Abstract

**Background:**

Air pollution may impair child growth and cognitive development, with potential markers including birth length and head circumference.

**Methods:**

The Household Air Pollution Intervention Network (HAPIN) trial was an open label multi-country-randomized controlled trial, with 3200 pregnant women aged 18–34 years (9–19 weeks of gestation) randomly assigned in a 1:1 ratio to receive liquefied petroleum gas (LPG) stove intervention compared to women continuing to cook with solid fuels for 18 months. Particulate matter ≤ 2.5 μm (PM2.5), black carbon (BC) and carbon monoxide (CO) 24-hour personal exposures were measured three times during pregnancy. Head circumference and length were measured < 24 h of birth. We conducted intention-to-treat and exposure–response analyses to determine the intervention effects and associations between household air pollution (HAP) exposure during pregnancy and head circumference, head circumference-for-gestational age Z-score, length, and length-for-gestational age Z-scores at birth. ClinicalTrials.gov.

**Results:**

Between May 2018, and Feb 2020, 3200 pregnant women were randomly assigned to intervention (n = 1593) and control groups (n = 1607) with 3060 births included in the analysis. There was a 71.9 % reduction in PM2.5 in the intervention group with similar reductions for BC and CO. Intention-to-treat analysis showed that the intervention did not affect head circumference (β = -0.01 cm, 95 %CI −0.11, 0.09), head circumference-for-gestational age Z-score (β = -0.01, 95 %CI −0.08, 0.07), or birth length (β = 0.14 cm, 95 %CI −0.01, 0.29) but did increase birth length-for-gestational age Z-score (β = 0.09, 95 %CI 0.01, 0.16). After covariate adjustment, exposure–response analysis revealed that each log-unit increase in BC was associated with a decrease in birth length-for-gestational age Z-score (β = -0.07, 95 %CI −0.13, −0.005). There was no evidence of hypothesized associations with PM2.5 or CO.

**Conclusion:**

An LPG intervention reduced HAP exposure during pregnancy but had minor effects on birth length-for-gestational age Z-score. Birth length-for-gestational age was only associated with BC.

**Clinical Trial Registration:** The study has been registered with ClinicalTrials.gov (Identifier NCT02944682).

## 1 Background

Approximately 3 billion people worldwide use solid fuels like wood, coal, and charcoal for cooking(Bonjour et al., 2013). Three quarters of the population in low- and middle-income regions, predominantly Africa, South and Southeast Asia, rely on these fuels,(Bonjour et al., 2013) leading to high levels of household air pollution (HAP), a significant cause of morbidity and mortality(Smith and Pillarisetti, 2017, Smith et al., 2014, Pope et al., 2017). HAP exposure during pregnancy has been linked to adverse birth outcomes, including low birthweight and impaired fetal growth, though findings have been inconsistent across studies (Amegah et al., 2014, Siddiqui et al., 2008, Gebremeskel Kanno and Hussen, 2021, Islam and Mohanty, 2021, Younger et al., 2022, Clasen et al., 2022, Wylie et al., 2014, Wylie et al., 2017). Systematic reviews and *meta*-analyses, mostly drawing from observational, or cross-sectional studies, has observed associations between prenatal air pollution exposure and restricted fetal growth measures, but clinical trials, including the HAPIN trial, have not found significant effects on birthweight (Clasen et al., 2022).

Head circumference and length at birth are crucial indicators of fetal growth and provide insights into early childhood development (Kozuki et al., 2015). Newborn head circumference is a measure of intrauterine brain growth, which is correlated with children’s future neurological and intellectual development (Kirchengast and Hartmann, 2020, Yusuf et al., 2019, Gale et al., 2006). Similarly, length at birth is a predictor of adult height(Eide et al., 2005), which in turn is associated with human and economic capital (Hoddinott et al., 2013). A systematic review and *meta*-analysis (Pun et al., 2021) demonstrated that prenatal exposure to air pollution resulted in a 19 % increased risk of postnatal stunting (WHO Child Growth Standards, 2009).

Interventions promoting the use of cleaner fuels, such as liquified petroleum gas (LPG), have been proposed to reduce exposures to HAP and to mitigate the health risks associated with solid fuel use. However, few studies have examined the effects of an LPG intervention during pregnancy on birth outcomes, including head circumference and length at birth, especially in low- and middle-income countries (Boamah-Kaali et al., 2021). Only one randomized controlled trial (RCT) of 1414 pregnant women in Ghana examined the relationship between stove type and head circumference and birth length (Boamah-Kaali et al., 2021, Quinn et al., 2021). This study found that infants in the LPG arm had decreased odds of smaller head circumference (odds ratio (OR) = 0.58, 95 % CI: 0.37, 0.92) (Boamah-Kaali et al., 2021). Same study reported that prenatal HAP exposure increased risk for reduced birth length [carbon monoxide (CO), OR = 1.17, 95 % CI: 1.01, 1.35 per 1-ppm increase; fine particulate matter (PM2.5) OR = 1.07, 95 % CI: 1.02, 1.13 per 10-μg/m3 increase] (Boamah-Kaali et al., 2021).

Despite this limited evidence base, research on HC and BL remains vital, particularly in low-resource settings where gestational age is often unassessed due to limited access to prenatal care and early ultrasounds. In such contexts, HC and BL, alongside birthweight, serve as important proxies for identifying small or growth-restricted infants.The Household Air Pollution Intervention Network (HAPIN) trial assessed the effects of an LPG stove and free fuel intervention on four primary outcomes, birthweight, child pneumonia and stunting, and blood pressure in adult women. Here we report on the intervention effects on exploratory outcomes, head circumference and length (including gestational age adjusted Z-scores) at birth. Additionally, we conducted corresponding exposure–response analyses to measure the associations between these exploratory outcomes and personal measures to fine particulate matter (PM2.5), black carbon (BC), and carbon monoxide (CO).

## 2 Methods

### 2.1 Study Design, setting and Participants

This study leverages data from the HAPIN trial, a multi-country randomized controlled trial with parallel assignment. Details of the study design, setting and participants are described elsewhere (Liao et al., 1987, Sambandam et al., 2020). Study sites in four countries (Guatemala, India, Peru, Rwanda) were selected based on formative research. HAPIN used a rolling recruitment process whereby each site enrolled 800 pregnant women (one per household) who met inclusion/exclusion criteria. Enrollment details and key characteristics of each study site have been published earlier (Barr et al., 2020, Clasen et al., 2020, Johnson et al., 2020). Pregnant women were eligible to participate in the study if they were (1) aged 18 to < 35 years; (2) lived in the study area; (3) used a biomass stove predominantly; (4) had a confirmed singleton, viable pregnancy between 9 to < 20 weeks gestation confirmed by ultrasound; (5) agreed to participate by providing their informed consent; and (6) were still pregnant at the time of randomization. Exclusion criteria included (1) all women who self-reported tobacco use, (2) planned to move permanently outside the study area, and (3) either used LPG stove predominantly or were likely to use LPG. Participants were followed until the child reached 1 year of age. Women were enrolled into the study between May 2018 and February 2020. Women were visited at home for a clinical assessment and 24-hour air pollution exposure monitoring at baseline (9–19 weeks), 24–28 weeks and 32–36 weeks gestation, to approximate three trimesters of pregnancy \(Checkley et al., 2024). Continuous personal monitoring was not feasible given the cost and logistics of monitoring 3,200 women at the same time, which required batteries to be recharged and filters to be replaced ever 24 h. Additionally, women had to wear vests or aprons for air pollution monitoring which would have been inconvenient, reducing compliance and making monitoring infeasible. We aimed to assess anthropometric measurements of newborns within 24 h of birth.

### 2.2 Intervention, randomization and masking

Households were randomly allocated to intervention or control arms after they consented to participate and baseline data were collected. Women were selected from four different countries, but within India and Peru there were separate sampling regions, such that these two countries were further divided into strata to ensure that the treatment and control groups were similar in each strata. A total of 10 randomization strata were implemented (India two sites; Peru six study sites, and Guatemala and Rwanda one site each). Additional details on sampled sites have been published earlier (Clasen et al., 2020). Participants were randomly assigned in a 1:1 ratio for each strata. The Data Management core developed the randomization scheme using a computer algorithm. Households in the intervention arm received a free LPG stove and continuous LPG supply during the study. Control households continued to use solid fuels for cooking. Given the nature of the intervention, field teams could not be masked to the intervention.

### 2.3 Ethics; trial registration; funding

The study protocol was reviewed and approved by institutional review boards (IRBs) or Ethics Committees at Emory University, Johns Hopkins University, Sri Ramachandra Institute of Higher Education and Research and the Indian Council of Medical Research – Health Ministry Screening Committee, Universidad del Valle de Guatemala and Guatemalan Ministry of Health National Ethics Committee, Asociación Benefica PRISMA, the London School of Hygiene and Tropical Medicine and the Rwandan National Ethics Committee, and Washington University in St. Louis. The study has been registered with ClinicalTrials.gov (Identifier NCT02944682).

### 2.4 Exposure measurement

Personal exposures to PM2.5, CO and BC were measured for 24-hours at each time point. The Enhanced Children’s MicroPEM (ECM, RTI International, Research Triangle Park) estimated personal exposures to PM2.5 from gravimetric samples collected on pre-weighed 15 mm Teflon filters (MTL,USA) using a 2.5 µm impactor at a flow rate of ∼ 0.3 L/minute. CO concentrations were measured using the Lascar EL-USB-CO300 (Lascar Electronics). Women wore these two lightweight devices built in a single vest or apron depending on cultural preferences in each country (e.g., apron in Guatemala and vest in India). Personal exposure to BC was estimated from PM2.5 filter samples using a Soot Scan Model OT21 Optical Transmissometer (Magee Scientific). Detailed personal exposure monitoring protocols and results have been published earlier (Johnson et al., 2020, Johnson et al., 2022).

### 2.5 Outcome measurement

Head circumference and birth length were measured by local trained field staff within 24 h of birth. Field staff were trained to maintain a standard operating procedure for measurements, using a seca 417 board (seca, Hamburg, Germany) to measure birth length and Gulick II tapes for head circumference. Two consecutive measurements were taken for birth length and head circumference and the measurements were rounded to the nearest 0.1 cm. A third measure was taken if the first two differed by > 0.7 cm for birth length and > 0.5 cm for head circumference. The two most proximate readings were averaged. Z-scores for head circumference-for-gestational age and birth length-for-gestational age were calculated based on INTERGROWTH-21st (International Fetal and Newborn Growth Consortium for the 21st Century) standards (Villar et al., 2014).

### 2.6 Statistical analysis

A statistical analysis plan was approved by the principal investigators prior to data analysis. Means and standard deviations were calculated for continuous variables; frequency and percentages were calculated for categorical variables, comparing the intervention and control groups, as well as for individual research sites.

To study the effect of the intervention on head circumference and length at birth (intention-to-treat analysis), we used linear regression models with treatment group as an independent variable, adjusted for the 10 randomization strata, using a similar approach has been used in prior publications form this trial (Checkley et al., 2024, Ye et al., 1979, Younger et al., 1987). No other baseline covariates were included in the final regression models. Separate models were created for each of the four outcomes of interest: head circumference (cm), head circumference-for-gestational age Z-score, birth length (cm) and birth length-for-gestational age Z-score. Subgroup analysis was conducted for the four countries.

We used linear regression models to quantify the association between PM2.5, BC and CO and continuous head circumference and length at birth, and their respective Z-scores as outcome variables (exposure–response analysis). HAP personal exposures during pregnancy were calculated as an average of all three measurements for controls. For participants in the intervention group, gestational exposures were calculated as a weighted average of pre- and post-intervention exposures, with the weights reflecting the proportion of gestational time spent in each period. The gestational age prior to the intervention served as the weight for the baseline exposure, when women in the intervention group were using the open fires, and the duration of gestation during the intervention served as the weight for the average post-baseline exposure when they were using their LPG stove. This approach accounted for changes in exposure due to the intervention and adjusted for the duration of exposure during pregnancy (Pillarisetti et al., 2023). All the HAP exposure variables (PM2.5, BC and CO) were natural log transformed to improve model fit.

Separate models were created for each outcome of interest with each exposure variable, 12 models in total. Models were adjusted for variables that might be associated with the outcomes. This included the 10 randomization strata, mother’s age, height, education, hemoglobin level, food insecurity and diet diversity score at baseline, nulliparity, number of people sleeping in the house, and child sex, as done in previous analyses (Balakrishnan et al., 2023). Child sex was not adjusted for models with Z-scores, as Z-score calculations already account for child sex. We conducted a complete case analysis. Only cases (i.e., individual observations) with complete data across all variables of interest are included in the analysis. Cases with any missing values are excluded entirely from the analysis. All analyses were conducted in R version 4.2.3 (R Core Team. R, 2021, Reporting, 2024).

### 2.7 Role of funding source

The funders and sponsors of this study did not have any role in the study design, collection, analysis or interpretation of the data. They did not interfere with report writing or the decision to submit this manuscript for publication.

## 3 Results

### 3.1 Study enrollment and Participant flow

**Fig. 1:**
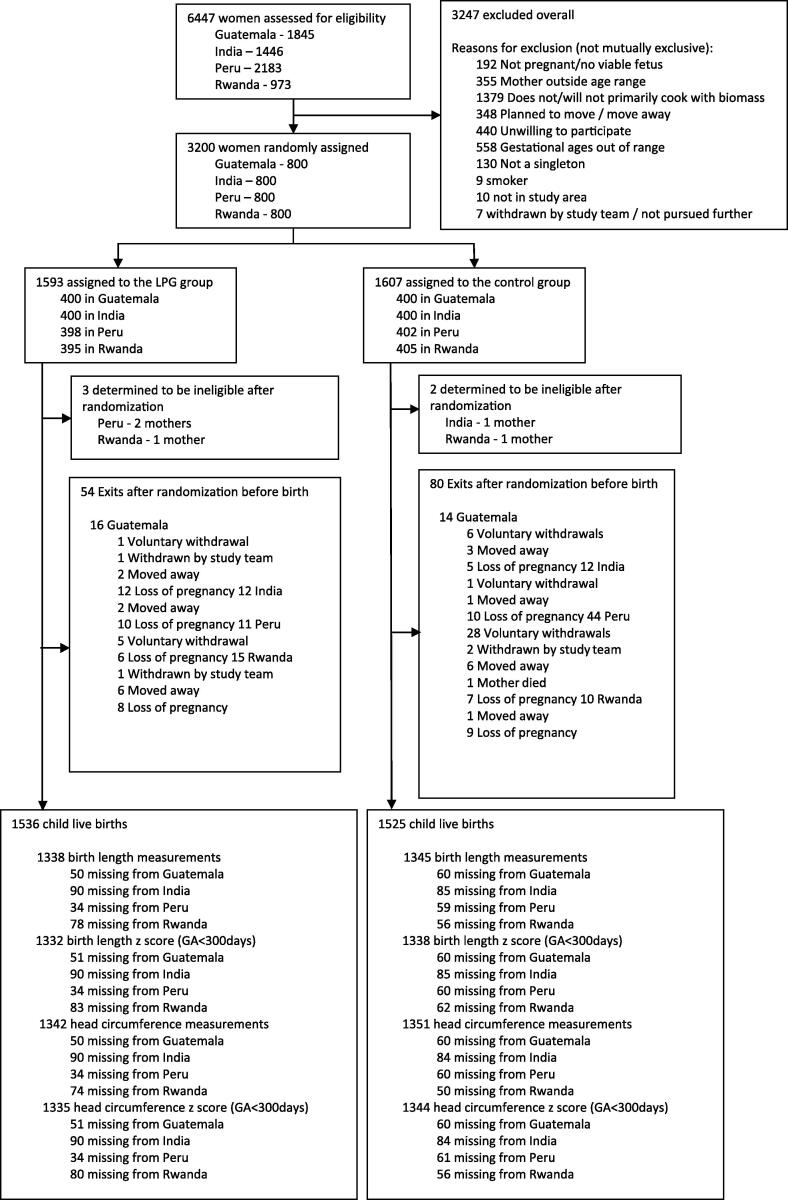
CONSORT Flow diagram of the participants enrolled in the HAPIN trial.

A total of 3200 women were enrolled in the study; five were found to be ineligible after randomization. We report on 3195 women (intervention = 1590; control arm = 1605) using the Consolidated Standards of Reporting Trials (CONSORT) flow diagram (Fig. 1). Among 3061 live births, 2475 (80.9 %) were measured by our staff at < 24 h of birth, and 125 (4.1 %) were measured after 24 h. The remaining live-born measurements, 83 (2.7 %) for length and 93 (3.0 %) for head circumference, could not be obtained by HAPIN staff during COVID-lockdown and were extracted from hospital records. There were 2683 (87.7 %) birth length measurements, 2670 (87.2 %) birth length-for-gestational age Z-scores, 2693 (88.0 %) head circumference measurements, and 2679 (87.5 %) head circumference-for-gestational age Z-scores. Z-scores were not calculated for infants with gestational age > 300 days per INTERGROWTH-21 criteria (Home • INTERGROWTH-21st. Accessed June 27, 2023), resulting in 13 missing birth length for gestational age Z-scores and 14 missing head circumference for gestational Z-scores.

### 3.2 Intervention coverage and Adherence

The intervention homes utilized LPG cookstoves almost exclusively, resulting in almost complete intervention coverage. The control homes continued using their conventional fuel and cookstoves (Clasen et al., 2022, Quinn et al., 2021). For the pregnant women from intervention arm, the median (Q1, Q3) elapsed time between randomization and the delivery of the intervention was 8 (5, 15) days; for Guatemala, it was 9 (5, 15); for India, it was 14 (9, 20); for Peru, it was 5 (2, 7); and for Rwanda, it was 11 (7, 20) days (Table 2). After randomization, an LPG stove was placed in 1156 households (72.9 %) during the intended 14-day window. The median time under intervention during pregnancy was 149 days (21 weeks, or around 5 months), and the median gestational age at the time of stove installation/first fuel supply was 17.9 (15.4, 20.6) weeks (Q1, Q3). In almost 86 % of intervention households, traditional cook stoves were either not used at all or used less than once each month of follow-up (Clasen et al., 2022, Quinn et al., 2021).

**Table 1:** Baseline Socio-Demographic Characteristics of Participating Women in the Trial (N = 3195).

	Control N = 1605	Intervention N = 1590	P-value
**Variable**	***Mean (SD)***	***Mean (SD)***	
**Mother height in cm**	152·1 (6·1)	152·3 (6·2)	0·30
**Mother’s body mass index (BMI)**	23·1(4·0)	23·3 (4·2)	0·23
**Mother’s hemoglobin level (g/dl)**	12·5 (1·9)	12·4 (1·9)	0·43
**Gestational age (weeks)**	15·3 (3·2)	15·5 (3·1)	0·07
	***N (%)***	***N (%)***	
**Mother’s age**			0·42
**<20 years**	209 (13·0)	189 (11·9)	
**20**–**24 years**	579 (36·1)	616 (38·7)	
**25**–**29 years**	517 (32·2)	500 (31·4)	
**30**–**35 years**	300 (18·7)	285 (17·9)	
**Nulliparous (missing = 6)**			0·04
**Yes**	1014 (63·3)	947 (59·7)	
**No**	589 (36·7)	639 (40·3)	
**Mother’s highest level of education (missing = 1)**			0·03
**No formal school**	558 (34·8)	481 (30·3)	
**Primary school**	533 (33·2)	558 (35.1)	
**Secondary/vocational**	514 (32·0)	550 (34·6)	
**Household food insecurity score (missing = 46)**			0·01
**Food secure**	863 (54·5)	930 (59·4)	
**Mild**	448 (28·3)	416 (26·6)	
**Moderate**	272 (17·2)	220 (14·0)	
**Mother’s minimum diet diversity (missing = 2)**			0·07
**Low**	906 (56·5)	890 (56·0)	
**Medium**	533 (33·2)	496 (31·2)	
**High**	165 (10·3)	203 (12·8)	
**Secondhand smoking (missing = 5)**			0·67
**No**	985 (61·5)	988 (62·2)	
**Yes**	617 (38·5)	600 (37·8)	
**Assets**			
**TV**	783 (48·8)	774 (48·7)	0·95
**Radio**	721 (44·9)	734 (46·2)	0·48
**Mobile Phone**	1395 (86·9)	1388 (87·3)	0·75
**Bicycle**	409 (25·5)	365 (23·0)	0·10
**Bank account**	628 (39·1)	697 (43·8)	0·01
**Child Sex (missing = 134)**			0·79
**Male**	787 (51·6)	800 (52·1)	
**Female**	738 (48·4)	736 (47·9)	
**Pregnancy Term (missing = 134)**			0·57
**Full Term**	1442 (94·6)	1446 (94·1)	
**Preterm**	83 (5·4)	90 (5·9)

**Table 2:** 24-hour personal exposure levels for fine particulate matter ≤ 2.5 μm in diameter (PM2.5 ug/m3), black carbon (BC ug/m3) and carbon monoxide (CO ppm) during pregnancy over time across the two treatment groups.

		**PM2.5 mean (SD),** **Median (IQR)**	**N**	**Within group % change from BL**	**BC mean (SD)** **Median (IQR)**	**N**	**Within group % change from BL**	**CO mean (SD)** **Median (IQR)**	**N**	**Within group % change from BL**
**Baseline** **(9**–**19 weeks)**	Control	110·9 (110·1), 83·1(45·9, 141·4)	1422	−	12.4 (9.4),10.8 (6.8, 15.5)	1272	−	2.3 (4.0),1.2 (0.5, 2.5)	1447	−
	Intervention	120·1 (134·8), 81·7 (45·9, 150·8)	1401	−	12.6 (11.0),10.6 (6.2, 15.3)	1267	−	2.7 (4.8),1.3 (0.5, 3.0)	1430	−
**24**–**28 weeks**	Control	104.4 (113.9),71.5 (38.6, 125.9)	1251	−5.9	11.1 (9.6),9.7 (5.3, 14.4)	1187	−10.5	2.3 (4.1) *,1.1 (0.4, 2.5)	1311	0
	Intervention	33.8 (33.1)*,24.1 (15.0, 39.5)	1285	−71.9	4.0 (5.5)*,2.7 (1.6, 4.7)	1226	−68.3	0.7 (1.5),0.2 (0.0, 0. 7)	1315	−74.1
**32**–**36 weeks**	Control	102.5 (107.7), 69.5 (36.5, 130.8)	1138	−7.6	11.1 (10.2),9.6 (5.2, 13.7)	1079	−10.5	2.2 (4.0)*,1.1 (0.3, 2.3)	1213	−4.4
	Intervention	35.8 (54.6)*,23.7 (15.0, 39.7)	1176	−70.2	4.3 (5.4)*,2.8 (1.7, 4.8)	1134	−65.9	0.7 (1.3),0. 2 (0.0, 0.8)	1227	−74.1

* P-value < 0.001based on the results of *t*-test for the difference between intervention and control groups.

### 3.3 Baseline demographic characteristics of Participants

Baseline demographic variables were generally similar across study arms (Table 1). Women in the intervention arm had slightly lower rates of nulliparity, higher educational attainment, and less food insecurity than women in control arm. Women across the four countries were also similar across arms except for India, where women in the control group had higher rates of nulliparity, and in Rwanda, where women in the intervention group had higher educational attainment and women in the control group were more food insecure and had lower diet diversity scores (Table S1).

### 3.4 Baseline and Follow-Up air pollutant measurements

**Fig. 2:**
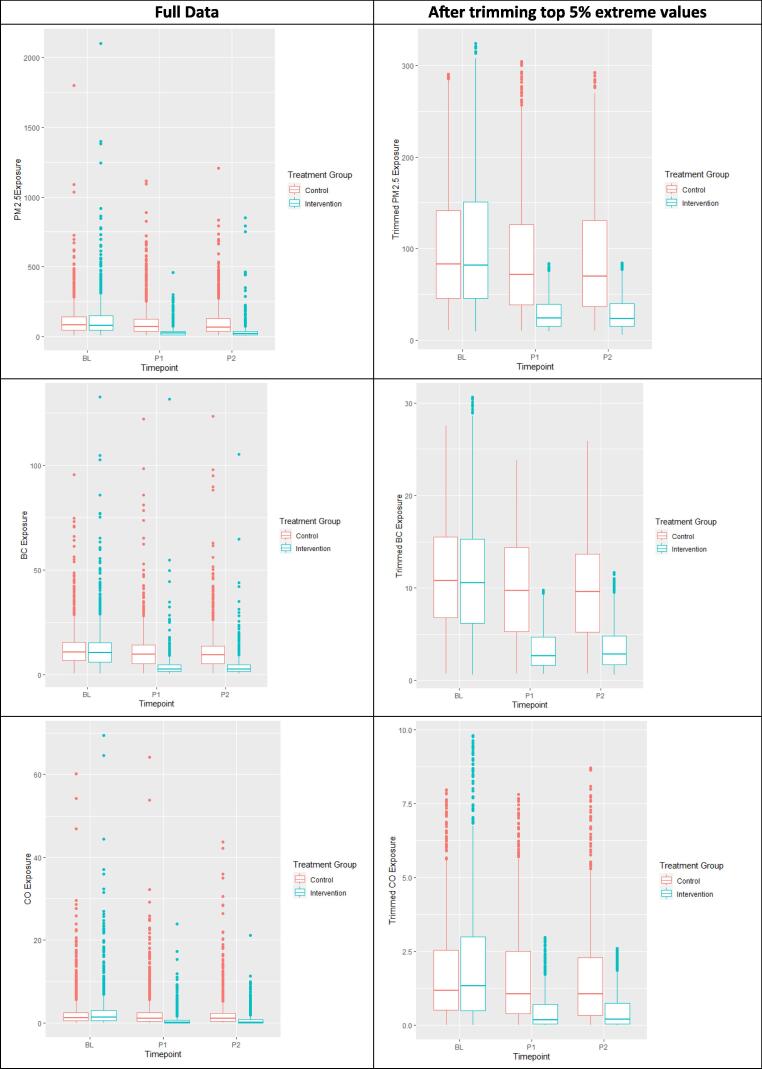
24-hour personal exposure levels for fine particulate matter ≤ 2.5 μm in diameter (PM2.5 ug/m3), black carbon (BC ug/m3) and carbon monoxide (CO ppm) during pregnancy over time across the two treatment groups.

While air pollutant measurements were comparable for both arms at baseline, there was a substantial reduction in all three pollutants among women in the intervention arm during follow-up (Table 2). PM2.5 was reduced from 120.1 μg/m^3^ at baseline to 35.8 μg/m^3^ at 32–36 weeks of gestation in the intervention arm. Similarly, BC was reduced from 12.6 μg/m^3^ at baseline to 4.3 μg/m^3^ at 32–36 weeks of gestation; and CO was reduced from 2.7 ppm at baseline to 0.7 ppm at 32–36 weeks of gestation in the intervention arm, with a significant difference between arms. Fig. 2 provides boxplots of the exposure shift among participating women at baseline and follow-up visits, by study arm. Among the four countries, Guatemala had the largest percent reduction (79.5 %) in PM2.5, whereas Peru had largest percent reduction (82.8 %) in BC and Rwanda had major percent reduction (80 %) in CO (Table S2). A more detailed description of exposure results has been previously reported (Johnson et al., 2022).

### 3.5 Intention-to-Treat analysis of birth outcomes

The intention-to-treat analysis of birth length, birth length-for-gestational age Z-score, head circumference and head circumference-for-gestational age Z-score demonstrated no effect of the intervention on outcomes except birth length-for-gestational age Z-score (Table 3). There was a 0.01 cm reduction in head circumference (β = -0.01, 95 %CI = -0.11, 0.09) and 0.01 reduction in head circumference-for-gestational age Z-score (β = -0.01, 95 %CI = -0.081, 0.07) in the intervention group, compared to the control group. Newborns in the intervention arm had a 0.14 cm increase in birth length (β = 0.14, 95 %CI = -0.01, 0.29) and 0.09 increase in birth length-for-gestational age Z-scores (β = 0.09, 95 %CI = 0.01, 0.16) compared to controls (Table 3). There were no significant differences between intervention and control arms on any outcomes in India, Rwanda, and Peru; in Guatemala there was a 0.19 increase in birth length-for-gestational age Z-scores (β = 0.19, 95 % CI = 0.04, 0.34) in newborns in the intervention arm.

**Table 3:** Regression models predicting the association of intervention with newborn birth length and head circumference (Intention to Treat Analysis).

	**Overall**	**Guatemala**	**India**	**Peru**	**Rwanda**
	**β (95 % CI)**	**β (95 % CI)**	**β (95 % CI)**	**β (95 % CI)**	**β (95 % CI)**
**Head circumference (cm)**	−0.01 (−0.11, 0.09)	0.10 (−0.10, 0.30)	−0.17 (−0.39, 0.06)	−0.05 (−0.23, 0.12)	0.09 (−0.11, 0.30)
**Head circumference Z-score**	−0.01 (−0.08, 0.07)	0.08 (−0.07, 0.23)	−0.14 (−0.31, 0.03)	−0.06 (−0.20, 0.08)	0.10 (−0.06, 0.26)
**Birth Length (cm)**	0.14 (−0.01, 0.29)	0.31 (−0.01, 0.64)	0.04 (−0.28, 0.35)	0.16 (−0.11, 0.44)	0.05 (−0.24, 0.35)
**Birth Length Z-score**	0.09* (0.01, 0.16)	0.19* (0.04, 0.34)	0.002 (−0.14, 0.15)	0.09 (−0.05, 0.23)	0.06 (−0.10, 0.21)

All models adjusted for the 10 randomization strata across 4 research sites.

* Significant at 95% level of confidence.

### 3.6 Exposure-Response analysis

Overall, the adjusted exposure–response analysis also showed no statistical association of PM2.5 or CO with any of the outcomes (Table 4). BC was only associated with birth length-for-gestational age Z-score. With each log-unit increase in PM2.5 exposure, there was a reduction of 0.02 cm in head circumference (β = -0.02, 95 %CI = -0.10, 0.05), 0.02 in head circumference-for-gestational age Z-score (β = -0.02, 95 %CI = -0.08, 0.03), 0.09 cm in birth length (β = -0.09 95 %CI = -0.20, 0.02) and 0.05 in birth length-for-gestational age Z-score (β = -0.05, 95 %CI = -0.11, 0.00). There was also a negative association between BC and head circumference (β = -0.05, 95 %CI = -0.13, 0.04), head circumference-for-gestational age Z-score (β = -0.06, 95 %CI = -0.12, 0.01), birth length (β = -0.09, 95 %CI = -0.21, 0.04) and birth length-for-gestational age Z-scores (β = -0.07, 95 %CI = -0.13, −0.005). With each log-unit increase in CO, there was an increase of 0.02 cm in head circumference (β = 0.02, 95 %CI = -0.02, 0.07), 0.02 in head circumference-for-gestational age Z-score (β = -0.02, 95 % CI = -0.02, 0.06), 0.02 cm in birth length (β = 0.02, 95 % CI = -0.05, 0.08) and 0.01 in birth length-for-gestational age Z-score (β = 0.01, 95 %CI = -0.02, 0.04).

**Table 4:** Regression models predicting the association of each exposure with birth length and head circumference for all participating women (exposure–response analysis).

	**PM 2.5 ug/m3**	**BC ug/m3**	**CO ppm**
	**β (95 % CI)**	**β (95 % CI)**	**β (95 % CI)**
**Head Circumference (cm)**	−0.02 (−0.10, 0.05)	−0.05 (−0.13, 0.04)	0.02 (−0.02, 0.07)
**Head circumference Z-score**	−0.02 (−0.08, 0.03)	−0.06 (−0.12, 0.01)	0.02 (−0.02, 0.06)
**Birth Length (cm)**	−0.09 (−0.20, 0.02)	−0.09 (−0.21, 0.04)	0.02 (−0.05, 0.08)
**Birth Length Z-score**	−0.05 (−0.11, 0.00)	−0.07* (−0.13, −0.005)	0.01 (−0.02, 0.04)

All models adjusted for 10 randomization strata, mother’s age at baseline, mother’s hemoglobin level at baseline, mother’s height at baseline, mother’s education, food insecurity index at baseline, diet diversity score at baseline, nulliparity, number of people sleeping in house, and child sex (child sex not included in models for z-score as z-scores are sex adjusted).

All exposure variables were natural log-transformed.

* Significant at 95% level of confidence.

## 4 Discussion

An LPG stove and free fuel intervention implemented across four rural, low-income settings had a small but statistically significant effect on increasing birth length-for-gestational age Z-score, but no effect on birth length, head circumference or head circumference-for-gestational age Z-scores. These results were mainly driven by effects observed among Guatemalan infants. Guatemala had an earlier baseline by 1–2 weeks (14.4 weeks gestation, compared to 16.1, 15.8 and 15.6 weeks in India, Peru and Rwanda, respectively), which may explain slightly greater effect on these infants. While Guatemala didn’t show the largest within group percent reductions in BC in the intervention group, Guatemala had the largest exposure contrast between the intervention and control groups (Johnson et al., 2022). In the exposure–response analysis, we found a significant negative association between BC and birth length-for-gestational age Z-score, but no other associations with head circumference, head circumference-for-gestational age Z-score, or birth length, and no association between PM2.5 or CO and any measured outcomes.

Findings from our study are consistent with a randomized intervention trial conducted in Ghana, which assessed the effect of an LPG stove on birth length and head circumference (Quinn et al., 2021). The authors found a significant difference in birth length with 1 ppm increase in CO exposure (β = -0.3 cm, 95 % CI = -0.6, −0.1 cm). Like ours, their results were not clinically significant. The difference in results may be explained by study setting and population; we present pooled results from four countries. Their study found no association between CO and head circumference. Other manuscripts from HAPIN investigating associations between HAP and birthweight, or stunting at 12 months, also did not find any associations (Clasen et al., 2022, Balakrishnan et al., 2023, Checkley et al., 2024). A recent HAPIN publication reporting on fetal outcomes did not find differences in averaged post-randomization Z scores for *in utero* head circumference (p-value = 0·04) (Checkley et al., 2024). It is worth noting that their threshold for statistical significance was 0·0125. This difference of findings might be explained by measurements assessed at different fetal stages via ultrasound, whereas our study reports on actual anthropometric measurements taken after birth. It is important to note that statistical significance does not necessarily translate into clinical significance. The clinical significance of differences in birth length and head circumference must be interpreted in the context of various influencing factors, including gestational age and other health-related conditions. These variables play a crucial role in understanding the implications of variations in these measures (McGlothlin and Lewis, 2014).

There are several possible explanations for our findings. First, other unmeasured sources of air pollution, both indoors and outdoors, may have important overall effects on newborn anthropometrics. A second explanation could be the timing of our intervention strategy. We enrolled women in the late-first to early-second trimester and LPG stoves were available in their second trimester. The preconceptual period and early pregnancy are important periods that may impact fetal growth and development. We know from literature that PM2.5 exposure during the pre-conception period is related to a higher risk of intellectual disability and small for gestational age in infants (Grineski et al., 2023, Chen et al., 2022) The first trimester of pregnancy is a critical period for fetal development, including head circumference and birth length(Pagano and Infant, 2023). During the first trimester, the baby's brain undergoes rapid development, including the formation of the neural tube, which eventually develops into the brain and spinal cord(Pagano and Infant, 2023). Similarly, during the first trimester, the baby's skeletal system begins to develop, including the formation of bones and joints. (Pagano and Infant, 2023) Early exposures to HAP could induce oxidative/nitrosative stress, increased inflammation, or epigenetic modifications leading to poor anthropometric outcomes (Saenen et al., 2019, Grevendonk et al., 2016, Sinharoy et al., 2020). Previous studies have documented that HAP has a more pronounced effect during the early stages of pregnancy (Grevendonk et al., 2016, Rossner et al., 2011). Our study missed much of the critical first trimester period, which could explain the lack of effect of the intervention on head circumference and birth length, despite significant measured 24-hour reductions in PM2.5, BC and CO exposures. Lastly, as head circumference and birth length were exploratory outcomes, it is acknowledged that the study was not adequately powered to detect statistically significant differences, even if such differences exist.

Our study has several strengths. This is the first multi-country trial to measure personal exposures to air pollutants and their effect on newborn anthropometrics. Results from our study are more generalizable than previous research, given the diverse sample. We assessed almost 81 % of study newborns within 24 h of birth using standard procedures, which is crucial for achieving accurate measurements. Our study found significant intervention effects on birth length-for-gestational age Z-scores in Guatemala, where 43.5 % of children under 5 are stunted (Data Warehouse and January 2024); these results may be indicative of true effects in a vulnerable population.

There were several limitations of our study. Firstly, father’s height may have a significant impact on child’s height (Sari and Sartika, 2021) but was not measured in our study. Secondly, we only adjusted for baseline levels of potentially time-varying covariates such as maternal hemoglobin, food insecurity and dietary diversity. Last and most importantly, we included two 24-hour measures of HAP during the latter half of pregnancy, which may have been insufficient to capture long-term exposures to air pollution from cooking and other exposures, like household garbage burning which may not have occurred on the day of measurement, vehicle pollution, and neighborhood-level HAP. Further, in our study, women, who knew they were being monitored, may have behaved differently. Participants in the intervention arm may have used undetected open fires to cook traditional foods, exposing them to pollutants outside the window of exposure measurement (Sambandam et al., 2020). Although we did include stove use temperature sensors on all observed biomass stoves in intervention homes, (Quinn et al., 2021, Johnson et al., 2022) long-term stove use behaviors are hard to capture. So, while we saw reductions in exposure measurements, these may not be a true representation of long-term cooking behaviors and exposure patterns, thus diluting the observed effect of the intervention.

Our study is one of few studies to assess the causal effects of HAP on birth length and head circumference--further inquiry is warranted. For future studies, enrollment during the early first trimester, and longer-term air pollution exposure assessment is recommended. While we know that continuous personal monitoring in these settings is not typically feasible, because most air pollution monitors are bulky and cause some degree of discomfort, earlier, and more frequent monitoring on a monthly basis might be feasible and offer a more complete understanding of women’s long-term exposures during pregnancy. Covariates like father’s height and additional time-varying variables, like maternal nutrition, should be considered. At present, HAPIN investigators continue to monitor long-term exposures and anthropometric outcomes in children enrolled in the follow-up study to the HAPIN trial.

## 5 Conclusion

Our intervention strategy successfully reduced HAP during pregnancy but had small or no effects on newborn head circumference and birth length. We found a small improvement in birth length-for-gestational age Z-scores among infants in the intervention arm, mainly driven by effects observed among Guatemalan infants. Birth length-for-gestational age was only associated with BC.

## 6 Article Summary

LPG stove intervention during pregnancy reduced household air pollution, with minor effects on birth length-for-gestational age Z-score; this outcome was inversely associated with black carbon.

### 6.1 What’s Known on This Subject

Nearly three-quarters of the population residing in low- and middle-income countries rely on solid cooking fuels. Household air pollution during pregnancy has been linked to adverse birth outcomes and can affect newborn anthropometrics, including head circumference and length at birth.

### 6.2 What This Study Adds

A liquefied petroleum gas (LPG) stove and continuous fuel intervention during pregnancy that reduced personal exposures to PM2.5, black carbon and carbon monoxide had small or no effects on head circumference and length at birth.

## 7 Submission Deleration

The work described has not been published previously except in the form of an abstract. This article is not under consideration for publication elsewhere. The article's publication is approved by all authors and tacitly or explicitly by the responsible authorities where the work was carried out. If accepted, the article will not be published elsewhere in the same form, in English or in any other language, including electronically without the written consent of the copyright-holder.

## 8 Role of Funder/Sponsor (if any)

The NIH example had no role in the design and conduct of the study. Bayer Healthcare example provided reagents for B-type natriuretic peptide testing.

Authors Contribution Statement

Ms. Hina Raheel conducted the data analysis, drafted the initial manuscript, and critically reviewed and revised the manuscript.

Drs. Sheela Sinharoy, Anaité Diaz-Artiga, Sarada S. Garg, Ajay Pillarisetti, Miles Kirby, Shirin Jabbarzadeh, Jennifer Peel, Jiantong Wang, Joshua Rosenthal and Ms. Amy Lovvorn helped in interpretation of data and critically reviewed and revised the manuscript.

Drs. Kalpana Balakrishnan, Marilu Chiang, Alexie Mukeshimana, Michael Johnson, John P. McCracken, Usha Ramakrishnan, Luke P. Naeher, Ghislaine Rosa, Thomas F. Clasen, Lisa M. Thompson helped conceptualize and design the study, acquisition of data, and critically reviewed and revised the manuscript.

All authors approved the final manuscript as submitted and agree to be accountable for all aspects of the work*.*

## Funding Sources

The HAPIN trial was funded by the U.S. National Institutes of Health (NIH; cooperative agreement 1UM1HL134590) in collaboration with the Bill & Melinda Gates Foundation (OPP113127).

## CRediT authorship contribution statement

**Hina Raheel:** Writing – original draft, Visualization, Validation, Software, Formal analysis, Data curation. **Sheela Sinharoy:** Writing – review & editing. **Anaité Diaz-Artiga:** Writing – review & editing, Project administration. **Sarada S. Garg:** Writing – review & editing. **Ajay Pillarisetti:** Writing – review & editing. **Kalpana Balakrishnan:** Writing – review & editing, Project administration. **Marilu Chiang:** Writing – review & editing, Conceptualization. **Amy Lovvorn:** Writing – review & editing, Project administration. **Miles Kirby:** Writing – review & editing, Conceptualization. **Usha Ramakrishnan:** Writing – review & editing, Funding acquisition. **Shirin Jabbarzadeh:** Writing – review & editing, Data curation. **Alexie Mukeshimana:** Writing – review & editing, Project administration. **Michael Johnson:** Writing – review & editing, Conceptualization. **John P. McCracken:** Writing – review & editing. **Luke P. Naeher:** Writing – review & editing, Conceptualization. **Ghislaine Rosa:** Writing – review & editing. **Jiantong Wang:** Writing – review & editing, Data curation. **Joshua Rosenthal:** Writing – review & editing. **William Checkley:** Writing – review & editing, Project administration, Funding acquisition, Conceptualization. **Thomas F. Clasen:** Writing – review & editing, Project administration, Funding acquisition, Conceptualization. **Jennifer L. Peel:** Writing – review & editing, Project administration, Funding acquisition, Conceptualization. **Lisa M. Thompson:** Writing – review & editing, Writing – original draft, Supervision, Project administration, Funding acquisition, Conceptualization.

## Declaration of competing interest

The authors declare that they have no known competing financial interests or personal relationships that could have appeared to influence the work reported in this paper.

## Data Availability

Data will be made available on request.
