# Supplementary material for: Effects of a liquefied petroleum gas stove and fuel intervention on head circumference and length at birth: A multi-country household air pollution intervention network (HAPIN) trial
Source: Environ Int. 2025 Jan;195:109211. doi: 10.1016/j.envint.2024.109211 (PMC11757157; doi:10.1016/j.envint.2024.109211)
Supplement: Supplementary Data 1 [file mmc1.docx]

**Supplementary Materials**: **Effects of a liquefied petroleum gas stove and fuel intervention on head circumference and length at birth: a multi-country household air pollution intervention network (HAPIN) trial**

| **Table S1. Baseline Socio-Demographic Characteristics of the Participating Women in the Overall Trial and Individual Research Sites.** | | | | | | | | | | | | | | | |
| --- | --- | --- | --- | --- | --- | --- | --- | --- | --- | --- | --- | --- | --- | --- | --- |
| **Variable** | **Overall** | | | **Guatemala** | | | **India** | | | **Peru** | | | **Rwanda** | | |
|  | Control  N=1605 | Intervention  N=1590 | P-value | Control  N=400 | Intervention  N=400 | P-value | Control  N=399 | Intervention  N=400 | P-value | Control  N=402 | Intervention  N=396 | P-value | Control  N=404 | Intervention  N=394 | P-value |
|  | ***Mean (SD)*** | ***Mean (SD)*** |  | ***Mean (SD)*** | ***Mean (SD)*** |  | ***Mean (SD)*** | ***Mean (SD)*** |  | ***Mean (SD)*** | ***Mean (SD)*** |  | ***Mean (SD)*** | ***Mean (SD)*** |  |
| **Mother height in cm** | 152.1(6.1) | 152.3 (6.2) | 0.30 | 148.1 (5.7) | 148.6 (5.0) | 0.32 | 151.2 (5.3) | 151.0 (5.9) | 0.58 | 152.6 (4.5) | 152.7 (4.6) | 0.77 | 156.1 (5.72) | 156.9 (6.0) | 0.06 |
| **Mother’s body mass index (BMI)** | 23.1  (4.0) | 23.3 (4. 2) | 0.23 | 23.7 (3.3) | 23.9 (3.4) | 0.46 | 19.6 (3.1) | 19.8 (3.3) | 0.35 | 25.8 (3.6) | 26.3 (3.6) | 0.04 | 23.5 (3.3) | 23.3 (3.5) | 0.52 |
| **Mother’s hemoglobin level (g/dl)** | 12.5(1.9) | 12.4 (1.9) | 0.43 | 12.9 (1.1) | 12.7 (1.0) | 0.08 | 10.4 (1.3) | 10.3 (1.2) | 0.30 | 14.3 (1.2) | 14.3 (1.3) | 0.98 | 12.4 (1.5) | 12.4 (1.6) | 0.73 |
| **Gestational age (weeks)** | 15.3(3.2) | 15.5 (3. 1) | 0.07 | 14.2 (3.1) | 14.4 (3.0) | 0.20 | 16.0 (3.1) | 16.1 (3.0) | 0.59 | 15.6 (3.5) | 15.8 (3.3) | 0.34 | 15.4 (2.7) | 15.6 (2.8) | 0.32 |
|  | ***N (%)*** | ***N (%)*** |  | ***N (%)*** | ***N (%)*** |  | ***N (%)*** | ***N (%)*** |  | ***N (%)*** | ***N (%)*** |  | ***N (%)*** | ***N (%)*** |  |
| **Mother’s age** |  |  | 0.42 |  |  | 0.65 |  |  | 0.97 |  |  | 0.19 |  |  | 0.83 |
| **<20 years** | 209 (13.0) | 189 (11.9) |  | 58 (14.5) | 64 (16.0) |  | 66 (16.5) | 62 (15.5) |  | 59 (14.7) | 40 (10.1) |  | 26 (6.4) | 23 (5.8) |  |
| **20-24 years** | 579 (36.1) | 616 (38.7) |  | 156 (39.0) | 168 (42.0) |  | 190 (47.6) | 192 (48.0) |  | 135 (33.6) | 151 (38.1) |  | 98 (24.3) | 105 (26.6) |  |
| **25-29 years** | 517 (32.2) | 500 (31.4) |  | 121 (30.2) | 110 (27.5) |  | 113 (28.3) | 117 (29.2) |  | 127 (31.6) | 130 (32.8) |  | 156 (38.6) | 143 (36.6) |  |
| **30-35 years** | 300 (18.7) | 285 (17.9) |  | 65 (16.2) | 58 (14.5) |  | 30 (7.5) | 29 (7.2) |  | 81 (20.1) | 75 (18.9) |  | 124 (30.7) | 123 (31.2) |  |
| **Nulliparous (missing=6)** |  |  | 0.04 |  |  | 0.39 |  |  | 0.03 |  |  | 0.94 |  |  | 0.60 |
| **Yes** | 1014 (63.3) | 947 (59.7) |  | 292 (73.0) | 281 (70.2) |  | 185 (46.4) | 155 (38.8) |  | 245 (61.1) | 239 (60.8) |  | 292 (72.5) | 272 (69.2) |  |
| **No** | 589 (36.7) | 639 (40.3) |  | 108 (27.0) | 119 (29.8) |  | 214 (53.6) | 245 (61.2) |  | 156 (38.9) | 154 (39.2) |  | 111 (27.5) | 121 (30.8) |  |
| **Mother’s highest level of education (missing=1)** |  |  | 0.03 |  |  | 0.79 |  |  | 0.15 |  |  | 0.06 |  |  | <0.001 |
| **No formal school** | 558 (34.8) | 481 (30.3) |  | 192 (48.0) | 189 (47.2) |  | 155 (38.8) | 130 (32.5) |  | 20 (5.0) | 15 (3.8) |  | 191 (47.3) | 147 (37.3) |  |
| **Primary school** | 533 (33.2) | 558 (35.1) |  | 152 (38.0) | 160 (40.0) |  | 111 (27.8) | 16 (29.0) |  | 103 (25.6) | 131 (33.2) |  | 167 (41.3) | 151 (38.3) |  |
| **Secondary/vocational** | 514 (32.0) | 550 (34.6) |  | 56 (14.0) | 51 (12.8) |  | 133 (33.3) | 154 (38.5) |  | 279 (69.4) | 249 (63.0) |  | 46 (11.4) | 96 (24.4) |  |
| **Household food insecurity score (missing=46)** |  |  | 0.01 |  |  | 0.57 |  |  | 0.80 |  |  | 0.43 |  |  | <0.001 |
| **Food secure** | 863 (54.5) | 930 (59.4) |  | 215 (54.3) | 225 (57.1) |  | 321 (80.7) | 324 (81.6) |  | 202 (51.1) | 210 (53.7) |  | 125 (31.7) | 171 (44.5) |  |
| **Mild** | 448 (28.3) | 416 (26.6) |  | 129 (32.6) | 126 (32.0) |  | 60 (15.1) | 54 (13.6) |  | 146 (37.0) | 128 (32.7) |  | 113 (28.7) | 108 (28.1) |  |
| **Moderate** | 272 (17.2) | 220 (14.0) |  | 52 (13.1) | 43 (10.9) |  | 17 (4.3) | 19 (4.8) |  | 47 (11.9) | 53 (13.6) |  | 156 (39.6) | 105 (27.3) |  |
| **Mother’s minimum diet diversity (missing=2)** |  |  | 0.07 |  |  | 0.67 |  |  | 0.76 |  |  | 0.14 |  |  | <0.001 |
| **Low** | 906 (56.5) | 890 (56.0) |  | 268 (67.0) | 279 (69.9) |  | 306 (76.7) | 315 (78.8) |  | 41 (10.2) | 46 (11.6) |  | 291 (72.2) | 250 (63.5) |  |
| **Medium** | 533 (33.2) | 496 (31.2) |  | 115 (28.6) | 104 (26.1) |  | 81 (20.3) | 73 (18.2) |  | 234 (58.2) | 203 (51.3) |  | 103 (25.6) | 116 (29.4) |  |
| **High** | 165 (10.3) | 203 (12.8) |  | 17 (4.2) | 16 (4.0) |  | 12 (3.0) | 12 (3.0) |  | 127 (31.6) | 147 (37.1) |  | 9 (2.2) | 28 (7.1) |  |
| **Secondhand smoking (missing=5)** |  |  | 0.67 |  |  | 1.00 |  |  | 0.63 |  |  | 0.33 |  |  | 0.52 |
| **No** | 985 (61.5) | 988 (62.2) |  | 236 (59.1) | 236 (59.1) |  | 276 (69.2) | 283 (70.8) |  | 217 (54.1) | 228 (57.6) |  | 256 (63.5) | 241 (61.3) |  |
| **Yes** | 617 (38.5) | 600 (37.8) |  | 163 (40.9) | 163 (40.9) |  | 123 (30.8) | 117 (29.2) |  | 184 (45.9) | 168 (42.4) |  | 147 (36.5) | 152 (38.7) |  |
| **Assets** |  |  |  |  |  |  |  |  |  |  |  |  |  |  |  |
| **TV** | 783 (48.8) | 774 (48.7) | 0.95 | 188 (47.0) | 169 (42.2) | 0.18 | 301 (75.4) | 291 (72.8) | 0.39 | 260 (64.7) | 247 (62.4) | 0.50 | 34 (8.4) | 67 (17.0) | <0.001 |
| **Radio** | 721 (44.9) | 734 (46.2) | 0.48 | 151 (37.8) | 153 (38.2) | 0.88 | 52 (13.0) | 57 (14.2) | 0.62 | 304 (75.6) | 289 (73.0) | 0.39 | 214 (53.0) | 235 (59.6) | 0.06 |
| **Mobile Phone** | 1395 (86.9) | 1388 (87.3) | 0.75 | 370 (92.5) | 361 (90.2) | 0.26 | 327 (82.0) | 328 (82.0) | 0.99 | 388 (96.5) | 378 (95.5) | 0.45 | 310 (76.7) | 321 (81.5) | 0.10 |
| **Bicycle** | 409 (25.5) | 365 (23.0) | 0.10 | 53 (13.2) | 45 (11.2) | 0.39 | 61 (15.3) | 60 (15.0) | 0.91 | 162 (40.3) | 147 (37.1) | 0.36 | 133 (32.9) | 113 (28.7) | 0.20 |
| **Bank account** | 628 (39.1) | 697 (43.8) | 0.01 | 98 (24.5) | 99 (24.8) | 0.94 | 359 (90.0) | 357 (89.2) | 0.74 | 86 (21.4) | 94 (23.7) | 0.43 | 85 (21.0) | 147 (37.3) | <0.001 |
| **Child Sex (missing=134)** |  |  | 0.79 |  |  | 0.47 |  |  | 0.91 |  |  | 0.63 |  |  | 0.61 |
| **Male** | 787 (51.6) | 800 (52.1) |  | 194 (50.3) | 203 (52.9) |  | 209 (54.0) | 208 (53.6) |  | 175 (48.9) | 195 (50.6) |  | 209 (53.0) | 194 (51.2) |  |
| **Female** | 738 (48.4) | 736 (47.9) |  | 192 (49.7) | 181 (47.1) |  | 178 (46.0) | 180 (46.4) |  | 183 (51.1) | 190 (49.4) |  | 185 (47.0) | 185 (48.8) |  |
| **Pregnancy Term (missing=134)** |  |  | 0.57 |  |  | 0.99 |  |  | 0.67 |  |  | 0.42 |  |  | 0.82 |
| **Full Term** | 1442 (94.6) | 1446 (94.1) |  | 361 (93.5) | 359 (93.5) |  | 363 (93.8) | 361 (93.0) |  | 343 (95.8) | 364 (94.5) |  | 375 (95.2) | 362 (95.5) |  |
| **Preterm** | 83 (5.4) | 90 (5.9) |  | 25 (6.5) | 25 (6.5) |  | 24 (6.2) | 27 (7.0) |  | 15 (4.2) | 21 (5.5) |  | 19 (4.8) | 17 (4.5) |  |

| **TableS2: 24-hour personal exposure levels for PM2.5 (ug/m3), BC (ug/m3) and CO (ppm) during pregnancy over time across the two treatment groups for individual research sites** | | | | | | | | | | | | | | |
| --- | --- | --- | --- | --- | --- | --- | --- | --- | --- | --- | --- | --- | --- | --- |
|  |  | **PM2.5 mean (SD),**  **Median (IQR)** | | **N** | **Within group % reduction from BL** | | **BC mean (SD)**  **Median (IQR)** | **N** | | **Within group % reduction from BL** | | **CO mean (SD)**  **Median (IQR)** | **N** | **Within group % reduction from BL** |
| **Guatemala** | | | | | | | | | | | | | | |
| **Baseline**  **(9-19 weeks)** | Control | 140.0 (133.4),  110.2 (63.3, 176.0) | | 373 | - | | 13.0 (7.5),  12.1 (9.1, 15.3) | 343 | | - | | 2.0 (3.7),  1.3 (0.5, 2.5) | 382 | - |
|  | Intervention | 153.0 (118.7),  122.3 (65.8, 200.2) | | 360 | - | | 13.4 (10.7),  11.7 (9.4, 14.8) | 332 | | - | | 2.1 (2.3),  1.4 (0.6, 2.8) | 375 | - |
| **24-28 weeks** | Control | 133.0 (116.1),  98.4 (58.7, 164.7) | | 339 | -5 | | 12.3 (6.6)*,  11.5 (8.1, 15.1) | 330 | | -5.4 | | 1.9 (2.2),  1.2 (0.5, 2.5) | 355 | -5 |
|  | Intervention | 31.3 (33.4)*,  23.3 (14.9, 36.2) | | 361 | -79.5 | | 4.9 (7.5),  2.7 (2.6, 5.3) | 359 | | -63.4 | | 0.5 (1.0),  0.1 (0.0, 0.5) | 363 | -76.2 |
| **32-36 weeks** | Control | 123.9 (99.5),  93.6 (53.6, 168.2) | | 317 | -11.5 | | 11.9 (7.1)*,  11.1 (8.4, 14.5) | 310 | | -8.5 | | 1.7 (2.1),  1.2 (0.4, 2.2) | 336 | -15 |
|  | Intervention | 33.4 (37.9)*,  23.8 (16.3, 38.5) | | 330 | -76.1 | | 5.0 (4.9),  2.9 (2.6, 5.7) | 326 | | -61.5 | | 0.6 (1.1),  0. 2 (0.0, 0.6) | 344 | -70 |
| **India** | | | | | | | | | | | | | | |
| **Baseline**  **(9-19 weeks)** | Control | 103.9 (100.1),  73.8 (46.7, 123.3) | | 358 | - | | 12.3 (9.9),  9.4 (5.5, 15.7) | 351 | | - | | 1.8 (3.6),  0.8 (0.3, 1.9) | 372 | - |
|  | Intervention | 127.1 (181.3),  78.4 (47.9, 142.9) | | 357 | - | | 13.5 (12.6),  9.8 (5.5, 16.4) | 348 | | - | | 1.7 (2.6),  0.9 (0.3, 2.1) | 373 | - |
| **24-28 weeks** | Control | 102.9 (114.5),  67.3 (38.9, 117.8) | | 311 | -1.0 | | 11.1 (10.3),  8.9 (4.5, 14.5) | 304 | | -9.8 | | 1.9 (3.2),  0.8 (0.2, 2.1) | 352 | -5.6 |
|  | Intervention | 39.2 (39.1)*,  28.7 (16.9, 45.5) | | 314 | -69.2 | | 3.5 (4.7)*,  2.1 (1.1, 3.6) | 305 | | -74.1 | | 0.4 (1.4)*,  0.0 (0.0, 0.3) | 347 | -76.5 |
| **32-36 weeks** | Control | 109.3 (123.5),  68.2 (36.3, 129.3) | | 284 | -5.2 | | 11.8 (12.3),  8.2 (4.4, 14.0) | 277 | | -4.1 | | 2.0 (3.8),  0.7 (0.1, 2.2) | 320 | -11.1 |
|  | Intervention | 36.5 (39.6)*,  25.3 (16.9, 41.8) | | 293 | -71.2 | | 4.2 (7.6)*,  2.5 (1.5, 4.1) | 289 | | -68.9 | | 0.4 (0.9)*,  0. 0 (0.0, 0.2) | 313 | -76.5 |
| **Peru** | | | | | | | | | | | | | | |
| **Baseline**  **(9-19 weeks)** | Control | 80.2 (94.8),  46.1 (15.2, 108.5) | | 327 | - | | 11.0 (11.6),  7.9 (2.3, 15.7) | 289 | | - | | 3.5 (5.0),  1.9 (0.7, 4.3) | 333 | - |
|  | Intervention | 89.5 (116.8),  53.3 (22.8, 111.2) | | 331 | - | | 11.6 (11.2),  8.7 (3.6, 15.5) | 307 | | - | | 4.4 (7.5),  1.9 (0.8, 4.8) | 326 | - |
| **24-28 weeks** | Control | 64.5 (104.2),  31.4 (14.5, 74.4) | | 269 | -19.6 | | 8.5 (10.1),  4.4 (1.6, 12.1) | 246 | | -22.7 | | 3.3 (6.7),  1.2 (0.3, 3.4) | 269 | -5.7 |
|  | Intervention | 20.8 (19.3)*,  14.6 (14.1, 23.1) | | 289 | -74.1 | | 1.9 (1.1)*,  1.6 (1.5, 1.6) | 268 | | -82.7 | | 1.4 (2.4)*,  0.7 (0.2, 1.8) | 264 | -60 |
| **32-36 weeks** | Control | 67.0 (124.7),  24.7 (14.5, 57.9) | | 219 | -16.5 | | 8.7 (13.8),  3.7 (1.6, 10.8) | 201 | | -20.9 | | 3.5 (6.3),  1.5 (0.5, 3.3) | 212 | 0 |
|  | Intervention | 28.1 (77.1)*,  14.6 (13.9, 18.6) | | 264 | -68.6 | | 2.0 (1.8)*,  1.6 (1.5, 1.6) | 242 | | -82.8 | | 1.2 (2.1)*,  0.6 (0.1, 1.3) | 250 | -72.7 |
| **Rwanda** | | | | | | | | | | | | | | |
| **Baseline**  **(9-19 weeks)** | Control | 115.6 (97.2),  94.0 (58.6, 144.8) | | 364 | - | | 13.0 (8.4),  11.8 (8.3, 15.6) | 289 | | - | | 2.0 (3.3),  1.1 (0.5, 2.0) | 360 | - |
|  | Intervention | 108.0 (98.4),  82.7 (47.8, 134.1) | | 353 | - | | 11.5 (8.8),  9.7 (6.6, 14.2) | 1267 | | - | | 3.0 (4.8),  1.2 (0.5, 3.0) | 356 | - |
| **24-28 weeks** | Control | 108.9 (109.8),  79.6 (48.5, 129.6) | | 332 | -5.8 | | 11.8 (10.6),  10.0 (7.0, 13.9) | 307 | | -9.2 | | 2.1 (3.3),  1.0 (0.4, 2.1) | 335 | -5 |
|  | Intervention | 43.1 (32.3)*,  33.6 (23.8, 50.0) | | 321 | -60.1 | | 5.3 (5.0)*,  4.2 (2.9, 5.9) | 294 | | -53.9 | | 0.6 (1.2)*,  0.2 (0.1, 0.7) | 341 | -80 |
| **32-36 weeks** | Control | 99.4 (77.9),  79.9 (45.9, 128.1) | | 318 | -14.0 | | 11.2 (7.1),  10.3 (6.4, 13.7) | 291 | | -13.8 | | 2.2 (3.6),  1.1 (0.4, 2.2) | 345 | -10 |
|  | Intervention | 45.0 (58.1)*,  28.2 (23.6, 49.1) | | 289 | -58.3 | | 5.5 (4.9)*,  3.8 (2.9, 6.2) | 277 | | -52.2 | | 0.6 (1.0)*,  0. 2 (0.1, 0.7) | 320 | -80 |
| *P-value <0.001based on the results of t-test for the difference between intervention and control groups | | | | | | | | | | | | | | |

| **Table S3: Distribution of outcomes by intervention and control arms** | | | |
| --- | --- | --- | --- |
|  | **Control (n=1605)** | **Intervention (n=1590)** | **P-value** |
| **Head circumference (cm)** | 33.59 (1.52) | 33.57 (1.60) | 0.725 |
| **Head circumference Z-score** | -0.17 (1.16) | -0.18 (1.20) | 0.832 |
| **Birth Length (cm)** | 47.53 (2.17) | 47.69 (2.15) | 0.069 |
| **Birth Length Z-score** | -0.94 (1.05) | -0.18 (1.20) | 0.017 |
